# Reevaluating the antiquity of the Palmrose site: Collections-based research of an early plank house on the northern Oregon Coast

**DOI:** 10.1371/journal.pone.0255223

**Published:** 2021-08-17

**Authors:** Gabriel M. Sanchez

**Affiliations:** Department of Anthropology, Michigan State University, East Lansing, Michigan, United States of America; Universidade Federal da Bahia, BRAZIL

## Abstract

Large-scale excavations conducted by Smithsonian Institution archaeologists and avocational archaeologists during the 1960s and 1970s at three sites in Seaside, Oregon, resulted in the recovery of a diverse range of material culture curated by multiple institutions. One site, known as Palmrose (35CLT47), provides compelling evidence for the presence of one of the earliest examples of a rectangular plank house along the Oregon Coast. Previous research suggests habitation of the Palmrose site occurred between 2340 cal BC to cal AD 640. However, recent research highlights significant chronometric hygiene concerns of previously reported radiocarbon dates for the Seaside area, calling into question broader regional chronologies. This paper presents a revised chronology for the Palmrose site based on 12 new accelerator mass spectrometry (AMS) radiocarbon dates of ancient cervid bones. I evaluate these new dates and previously reported radiocarbon dates from the site, applying chronometric hygiene assessments and Bayesian statistics to build a refined chronology for the Palmrose site. Calibration of the 12 AMS radiocarbon dates suggests an initial occupation range from 345−55 cal BC and a terminal occupation range from cal AD 225−340−. Bayesian modeling of the Palmrose sequence suggests initial occupation may have spanned from *195−50 cal BC* and the terminal occupation from cal *AD 210−255*. Modeling suggests the maximum range of occupation may span from *580−55 cal BC* to *cal AD 210−300* based on the start and end boundary calculations. Bayesian modeling of radiocarbon dates directly associated with the plank house deposits suggests the plank house’s occupation may have spanned from *160−1 cal BC* to *cal AD 170−320*. The new radiocarbon dates significantly constrain the Palmrose habitation and alter regional chronologies.

## Introduction

In recent years, there has been a growth in the number of museum-based research studies that revisit and reanalyze archaeological legacy collections [1–12]. These projects have investigated a wide range of issues, including colonialism, environmental studies, gender, human subsistence, museum curation practices, and several other topics. Researchers are increasingly revisiting legacy collections and conducting new excavations at extant sites that apply modern excavation and sampling standards to revise site-specific and regional chronologies and earlier interpretations [9, 13–18]. In certain instances, archaeological sites are no longer accessible for new excavations due to site destruction, modification, permitting processes, heritage conservation practices, or concerns raised by stakeholders. In others, unanalyzed and understudied museum collections exist for the site(s) and do not warrant further excavations on sensitive, finite, and nonrenewable cultural resources. In these circumstances, museum collections offer an exceptional opportunity to contribute important new information regarding archaeological sites that can confirm, revise, and refine previously reported chronologies and interpretations for specific sites or broader archaeological regions.

Significant advances in radiocarbon dating have occurred since the inception of the method facilitated by accelerator mass spectrometry (AMS), increasing the accuracy and precision of radiocarbon dating measurement. Furthermore, advancements in sample preparation and pretreatment, smaller sample sizes required for dating, standardization of laboratory protocols, compound-specific analyses, refinement of calibration curves, improved statistical analyses, and a deeper understanding of reservoir effects continue to advance the method and its application. These developments have resulted in a critical reappraisal of previously reported archaeological chronologies, often through the application of site and region-specific chronometric hygiene assessments to ensure that radiocarbon samples are reliable for chronology building. Numerous studies have shown that various preceding radiocarbon dating projects frequently lack chronometric hygiene assessments and suffer from other biases. These biases commonly result from sampling long-lived rather than short-lived organisms, selecting mixed samples rather than single entities, lack of proper sample pretreatment procedures, estimated rather than measured δ^13^C and δ^15^N isotopic values, and dating samples of ambiguous cultural association [19–26].

Along the northern Oregon Coast, recent AMS radiocarbon dating of cervid bones at the Par-Tee site (34CLT20) by Sanchez and colleagues [9] significantly revised the Par-Tee chronology through the application of site-specific chronometric hygiene assessments to previously reported radiocarbon dates from the 1960s and 1970s and Bayesian statistical modeling. Sanchez and colleagues [9] found that radiocarbon measurements from the 1960s and 1970s at the Par-Tee site derive from composite or bulk samples of unidentified charcoal and shell and bone. Many samples were not appropriately pretreated to remove potential contaminants, and often, samples were not corrected for δ^13^C isotopic fractionation [19, 27–29]. Instead, δ^13^C isotopic ratios were estimated rather than measured, making these older dates problematic for building chronologies. These biases in the radiocarbon data are significant considering recent analyses of the Par-Tee site museum assemblage investigating ancient fishing practices, potential whaling events, and the use of cetaceans, sea mammals, and terrestrial mammals more broadly [30–35]. The lack of accurate radiometric measurements for the Par-Tee site places these studies in chronological limbo resulting in significant uncertainties regarding how the timing of the human activities identified at Par-Tee interdigitates with the Palmrose site and other sites and practices regionally.

Previous radiocarbon dating of a nearby archaeological site known as Palmrose (35CLT47) with evidence of an early plank house suggests the Palmrose site was inhabited millennia before Par-Tee. Because the majority of Palmrose radiocarbon samples were obtained by the same researchers, analyzed using the same methods, and samples processed by the same laboratory—the Smithsonian Institution Radiocarbon Laboratory (SI)—as Par-Tee, there are significant questions about their hygiene and the chronology’s reliability.

In this paper, I present the results of recent AMS radiocarbon dating and Bayesian analysis for the Palmrose site, a large village site that produced a sizeable and diverse material culture record that includes formal tools, faunal remains, and early evidence of fully- to semi-sedentary lifeways along the Oregon Coast. In this study, I primarily selected culturally modified cervid remains for radiocarbon dating, including cut marked elk (*Cervus*) and deer (*Odocoileus* sp.) bones that exhibit evidence of human processing. One exception is a single elk premolar/molar fragment from an excavation level that lacked other diagnostic postcranial cervid specimens and direct evidence of human processing. Previous research suggests that elk and deer dominate the Palmrose terrestrial mammal assemblage [30, 31] and represent the primary raw material in the bone and antler tool assemblage [9, 34, 36]. The Bayesian analysis of the new AMS radiocarbon dates for the Palmrose site sequence will assist forthcoming museum-based studies through the construction of a refined site chronology with relevance to broader regional chronological frameworks and provides an important context for enhancing the interpretation of existing collections and increasing their broader value to the scientific community [37–39].

## Background

**Fig 1:**
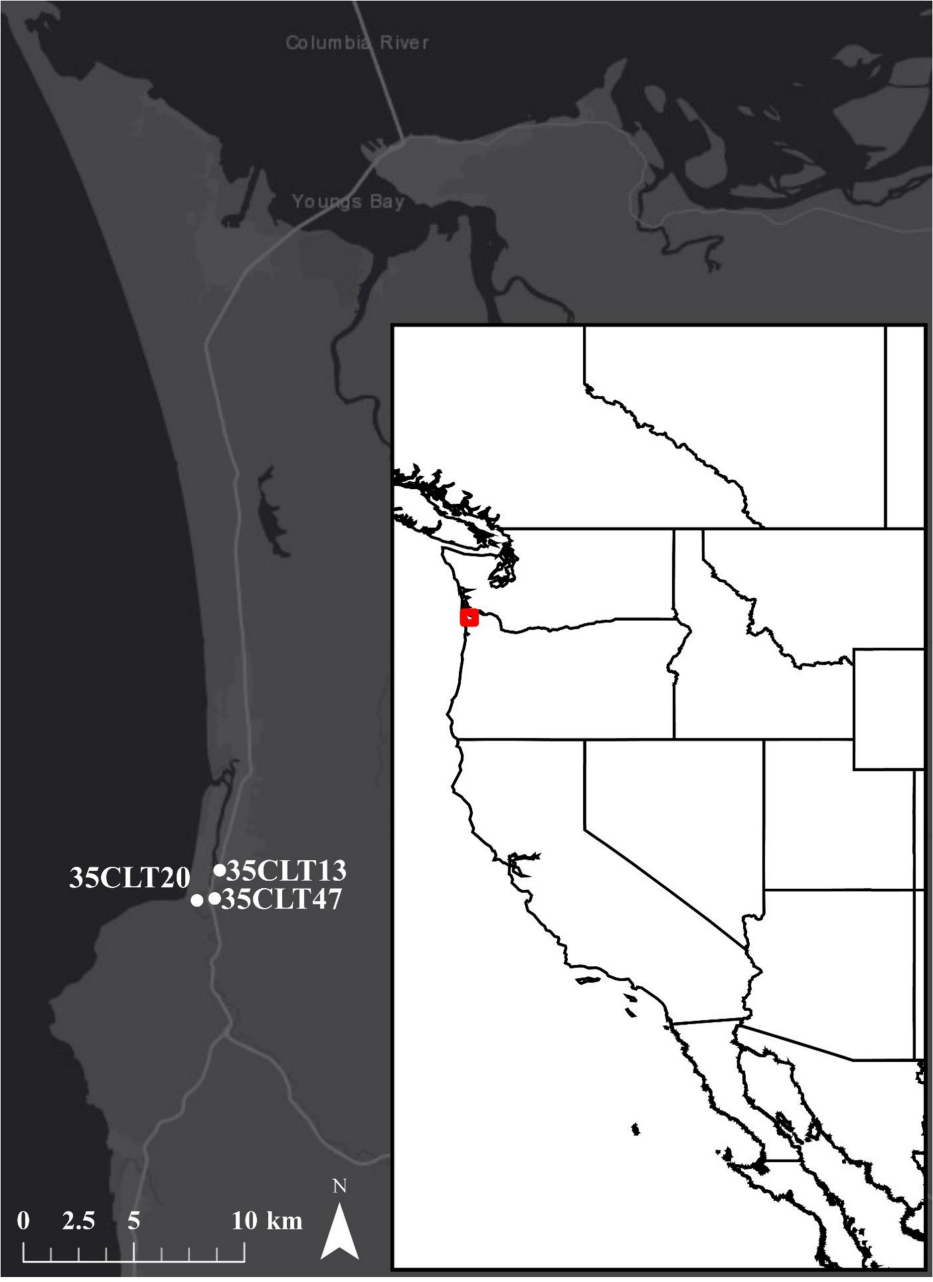
Overview of the northern Oregon Coast and the location of the Palmrose (35CLT47), Par-Tee (35CLT20), Avenue Q (35CLT13) sites.

Large-scale archaeological excavations along the northern Oregon Coast were conducted by George Phebus, a collections assistant in the Department of Anthropology, Smithsonian Institution, and avocational archaeologist Robert Drucker from 1967 to 1977 [40, 41]. Together Phebus and Drucker excavated three significant sites in Seaside, Oregon, specifically the Palmrose, Par-Tee, and Avenue Q (35CLT13) sites (Fig 1). Excavations resulted in the recovery of a diverse range of material culture currently curated by two institutions, including the National Museum of Natural History (NMNH), Smithsonian Institution, and the Museum of Natural and Cultural History (MNCH), University of Oregon [9]. However, many formal artifacts from the sites remain in possession of private collectors who participated in the initial excavations [42]. Phebus and Drucker note that Par-Tee and Palmrose each measured over 65 m in length with deposits of at least 1.4 m but up to 3.0 m in depth. The Avenue Q site lies beneath residential structures, yards, and roads but yielded stratified and undisturbed deposits.

Based on evidence from field notes curated by the MNCH and the National Anthropological Archives, Smithsonian Institution, Phebus and Drucker excavated at least 227 5 x 5 ft wide excavation units at the Palmrose site, 256 5 x 5 ft units at Par-Tee, and a single 5 x 5 ft test unit at Avenue Q. All excavation units were dug in 1 ft arbitrary levels—each assigned a numeric number from top to bottom—with the recovery of materials from excavated sediments screened over ¼ in. sieves. According to Phebus and Drucker’s estimates, excavations at Palmrose and Par-Tee may have totaled ~1415 m^3^.

### Palmrose site excavations 1967−1988

**Fig 2:**
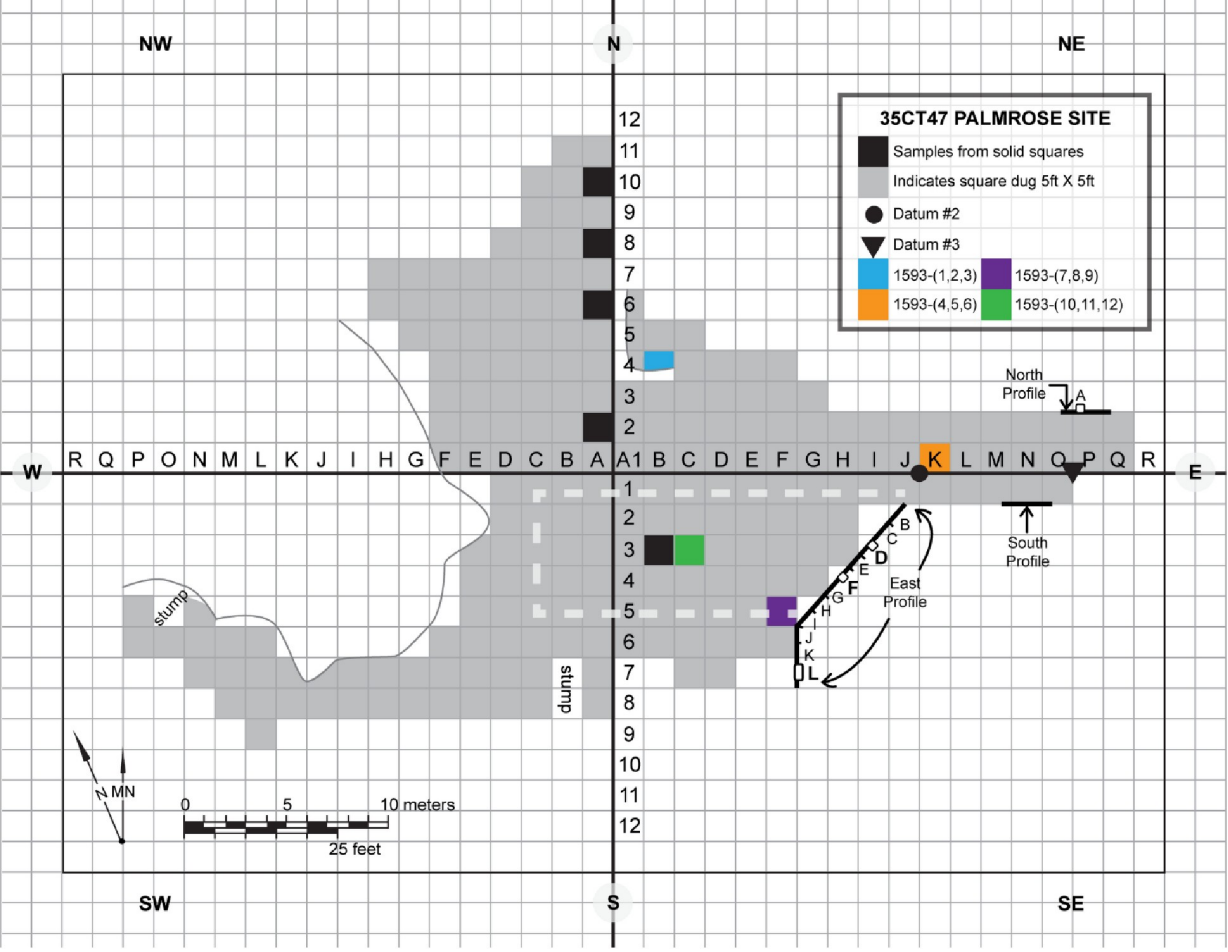
Grid map of the Palmrose site depicting excavation units sampled for radiocarbon dating (colored units) and the MNCH profiles from 1988. Grey units represent the extent of Phebus and Drucker’s excavations. Adapted from Connolly (42) with permission from the Museum of Natural and Cultural History, original copyright 1992.

Among the Seaside sites, Palmrose provides compelling evidence for the presence of a rectangular plank house, the earliest reported and known example of an ancient plank house along the Oregon Coast [40–43]. According to Phebus and Drucker’s field notes and reports, the site’s western portion was significantly impacted by looting activities and contained largely unstratified deposits [40, 41]. However, the eastern portion of the site appeared to be mostly intact with stratified deposits. Phebus and Drucker’s [40, 41] reports and field notes suggest they encountered a rectangular house feature, most likely a plank house, possibly measuring 6 m in width and 12 m in length, on the eastern portion of the site where they focused the majority of their excavation efforts (Fig 2). According to these records, the house included multiple superimposed sand-lined hearth features. Radiocarbon dates obtained in the 1960s and 1970s, primarily on unidentified charcoal, suggest the house was inhabited for millennia with at least three house rebuilding events [40, 41]. Phebus and Drucker’s interpretations of these dates suggest the site was inhabited in three significant episodes, with the earliest occurring from ~700−600 cal BC, an intermediate occupation from ~300−200 cal BC, and the terminal occupation from ~cal AD 200−300 [40–43].

In the summer of 1988, subsequent testing of the Palmrose site was conducted by MNCH archaeologists to establish the boundaries of a highway right-of-way for a proposed alteration to the local highway [42]. The MNCH field crews were able to relocate Phebus and Drucker’s former excavation units while confirming and establishing the site boundaries through the placement of seventeen 20 cm diameter auger probes, two 50 cm square test units, and one l x 1 m square test unit. The fieldwork confirmed earlier reports of extensive disturbance to the western section of the site. Given the disturbance level on the western portion of the site, MNCH archaeologists abandoned that section’s excavations. Subsequently, they focused their efforts on the site’s eastern segment, opening three vertical profiles of undisturbed midden deposits from Phebus and Drucker’s excavation units (Fig 2) [42].

Connolly [42] summarized that two profiles designated North and South both measured two meters in length and revealed stratified and intact deposits related to the plank house occupation (see Fig 2). However, Connolly terminated the South Profile’s excavation due to the presence of human remains in the basal deposits. Next, a 50 cm x 50 cm column sample designated as Unit A was excavated into the North Profile of Phebus and Drucker’s excavation block and northeast of the plank house. Lastly, a third profile designated as the East Profile measured 11 m in length and exposed a cross-section at the plank house feature’s eastern edge. All midden constituents were recovered using 1/8 in. mesh screens. The 1988 excavations confirm several factors originally reported by Phebus and Drucker [40, 41]. First, the house appears to have a well-defined bench along the north wall. Second, a central fire hearth provides evidence for a series of four superimposed sand-lined hearths from subsequent occupations, each marked "Sand or Ash/Sand" in field notes, reports, and profiles. Third, evidence suggests infilling occurred after site abandonment following each occupation [42].

### Palmrose radiocarbon dating 1967−1988

Previous research suggests habitation of Palmrose occurred between 2340 cal BC to cal AD 640 [40–42]. The bulk of the Palmrose site’s available radiocarbon dates derive from assays on charcoal samples submitted by Phebus and Drucker and processed by the Smithsonian Institution Radiocarbon Laboratory in the 1960s and 1970s. Table 1 presents the 19 radiocarbon assays. Before including or excluding these previously reported dates from Bayesian modeling, I applied the following chronometric hygiene assessments, which were previously used by Sanchez and colleagues [9] at the Par-Tee site, to evaluate each sample’s reliability: 1) are the samples derived from identified or unidentified charcoal, and do they represent bulk samples or individual specimens; 2) are samples from long-lived or short-lived organisms; 3) were sample pretreatment procedures conducted to remove potential contaminants; 4) were samples corrected for δ^13^C isotopic fractionation; 5) are samples accurately dating the event of interest or stated otherwise is there ambiguity regarding the association of the sample with cultural remains, deposits, and events of interest.

**Table 1:** Previously reported radiocarbon (^14^C) dates for the Palmrose site. Lab numbers beginning with Smithsonian Institution Radiocarbon Laboratory (SI) represent Phebus and Drucker samples, while Connolly submitted samples to Beta Analytic Inc. (Beta).

^14^C Lab Number	Provenience	Material	Pretreatment	Conventional ^14^C Age BP	cal BC/AD (95.4% CI)
**SI_612**	NWA2-4	Charcoal	---	1760 ± 50	AD 200−420
**SI-613**	NWA2-5	Charcoal	---	1650 ± 100	AD 210−640
**SI-614**	NWA6-6	Charcoal	---	1640 ± 100	AD 220−640
**SI-582**	NWA2-2	Charcoal	NaoH, HCl	2410 ± 110	800−200 BC
**SI-582R**	NWA2-2	Charcoal	NaoH, HCl	2610 ± 90	980−420 BC
**SI-583**	NWA2-6	Charcoal	NaoH, HCl	2260 ± 100	750−40 BC
**SI-584**	NWA6-7	Charcoal	NaoH, HCl	2620 ± 90	990−420 BC
**SI-584R**	NWA6-7	Charcoal	NaoH, HCl	3840± 150	2860−1880 BC
**SI-585**	NWA6-8	Charcoal	NaoH, HCl	2180 ± 80	400−10 BC
**SI-586**	NWA10-10	Charcoal	NaoH, HCl	2180± 100	420−70 BC
**SI-2385**	NE2C-6	Charcoal	NaoH, HCl	2495 ± 65	790−410 BC
**SI-2386**	NE2B-7	Charcoal	NaoH, HCl	2475 ± 65	780−410 BC
**SI-2387**	NE1D-5	Charcoal	NaoH, HCl	2490 ± 65	790−410 BC
**SI-2388**	NE1D-6	Charcoal	NaoH, HCl	2380 ± 65	770−260 BC
**SI-3229**	SE3B-3	Charcoal	NaoH, HCl	1765 ± 65	AD 120−420
**SI-3230**	SE3B-5	Charcoal	NaoH, HCl	1840 ± 65	AD 20−370
**SI-3231**	SE3B-7	Charcoal	NaoH, HCl	1830 ± 70	AD 30−410
**SI-3232**	SE3B-9	Charcoal	NaoH, HCl	2135 ± 65	380 BC−AD 10
**SI-3233**	SE3B-10	Charcoal	NaoH, HCl	2565 ± 70	890−420 BC
**Beta-28848**	Unit A-6	Charcoal	NaoH, HCl	1760 ± 60	AD 130−420
**Beta-28849**	Unit A-9	Charcoal	NaoH, HCl	2270 ± 100	750−40 BC
**Beta-28852**	Unit D-4	Charcoal	NaoH, HCl	3650 ± 100	2340−1740 BC
**Beta-28853**	Unit F-18	Charcoal	NaoH, HCl	2060 ± 100	370 BC−AD 210

Chronometric hygiene assessments have been applied in archaeological studies to assess the reliability of radiocarbon dates for various regions throughout the world [19–26]. While the criteria applied in chronometric hygiene assessments vary between regions due to differences in preservations biases, excavations practices, radiocarbon sample selection, and freshwater and marine reservoir effects, these assessments are applied to ensure that reported radiocarbon dates reflect the cultural phenomena of interest and to identify which samples should be included in analyses or excluded. Therefore, the chronometric hygiene criteria applied in this study seek to mitigate potential biases from historically reported dates that primarily derive from unidentified charcoal samples and composite charcoal samples [9, 42].

The chronometric hygiene assessments developed by Sanchez and colleagues [9] and applied in this study to evaluate the reliability of the 19 previously reported radiocarbon dates by Phebus and Drucker reveal numerous issues. First, the charcoal samples submitted by Phebus and Drucker represent large composite samples of wood, often combining separate entities in one sample. These findings are consistent with their use of composite samples at the Par-Tee site [9]. As Ashmore demonstrates [19], composite samples of wood are unreliable due to the combination of separate entities, resulting in the dating of multiple events rather than more discrete cultural activities. Second, in Pacific Northwest rainforests, long-lived trees and drift logs were a common fuel source, so dates of multiple unidentified charcoal fragments are likely significantly offset by in-built age and/or the old wood effect [9, 36, 44, 45]. Third, samples that lack stable carbon isotope measurements are prone to inaccuracies [28].

Based on archival records, δ^13^C isotopic values for all Smithsonian Institution radiocarbon samples from Palmrose were estimated rather than measured, raising uncertainties about correcting these dates. Fourth, several of these dates have large standard deviations (≥100 years) that result in large calibration ranges limiting their potential to provide the chronological data required to define and constrain the cultural events of interest. Fifth, the laboratory reanalyzed two samples submitted by Phebus and Drucker, and in each instance, discrepancies exist between the dates reported. For instance, SI-584 and SI-584R have conventional radiocarbon ages of 2620 ± 90 and 3840 ± 150, Table 1. When calibrated at 2-sigma in OxCal 4.4 using the IntCal20 calibration curve [46, 47], the dates span from 990−420 cal BC and 2860−1880 cal BC. To a lesser degree, SI-582 and SI 582R have conventional radiocarbon ages of 2410 ± 110 and 2610 ± 90, respectively, Table 1. When calibrated at 2-sigma, the dates span from 800−200 cal BC and 980−420 cal BC. For all these reasons, in re-examining the potential age range for Palmrose site human occupation and applying the chronometric hygiene assessments developed for this study, I exclude all dates previously reported by Phebus and Drucker [20, 23–26, 48, 49].

In addition to the dates compiled by Phebus and Drucker, four additional radiocarbon dates for the site were collected by MNCH staff and submitted to Beta Analytic Inc. following the 1988 field project. Similar to the dates reported by Phebus and Drucker, the radiocarbon dates reported by Connolly [42] were not corrected for δ^13^C isotopic fractionation, and the majority—three out of the four—is either derived from ambiguous contexts or lack sufficient data reporting to assess their cultural association fully. For example, sample Beta-28852 is derived from below the shell midden deposits of the Palmrose site beneath two clay lenses in humic loam [42]. The charcoal sample was derived from a charcoal-rich sandy loam near a whale bone fragment. However, given the lack of a well-defined association with cultural materials based on the lack of stone tools, shell midden, or other cultural items, it is unclear if the charcoal-rich sandy loam and whale bone represent natural background materials or if they were deposited through human agency. Given the ambiguous cultural association, the date is excluded. Of the remaining three samples submitted by Connolly, the provenience for two (Beta-28848 and Beta-28849) are not adequately reported. Beta-28853 represents charcoal from deposits Connolly interpreted as a sand-line hearth. Nevertheless, the samples analyzed represent unidentified charcoal, which may derive from long-lived organisms and includes multiple entities [19]. Applying the chronometric hygiene standards developed for this study and applied to other sites in the region [9], given the ambiguous and unreported cultural context for most of the samples, the use of unidentified charcoal, possibly from long-lived organisms, and lack of δ^13^C isotopic fractionation measurements, I excluded the four dates reported by Connolly [42] from chronological modeling.

## Methods and materials

The MNCH curates the Palmrose collections and materials sampled in this study. Twelve culturally modified elk and deer bone samples from four excavation units, including NE1K, NE4B, SE3C, and SE5F, were sampled in this analysis (Fig 2). The only specimen that lacked diagnostic cultural modification is sample 1593–5, an elk premolar/molar selected from an excavation level where other diagnostic postcranial deer and elk elements were not identified. These four units were selected as previous research by Phebus and Drucker [40, 41] and Connolly [42] places two of the four units, SE3C and SE5F, within the rectangular plank house feature. Units NE1K and NE4B lie to the north of the plank house feature but within possibly intact and stratified midden deposits. However, it is important to note that a portion of NE4B was impacted by looters affecting the integrity of the southern portion of the unit. Nonetheless, the selection of units from within and outside the plank house provides the opportunity to accurately date the overall Palmrose occupation and duration of the plank house habitation.

I selected three samples per unit, each from distinct arbitrary excavation levels and different strata within each excavation unit. In general, I selected samples from the basal, intermediate, and upper deposits of the unit to measure the site occupation’s extent. Where possible, I attempted to select specimens that did not crosscut strata noted by the original excavators. However, given the complex stratigraphy of the site, that was not always possible. I used a Dremel^TM^ drill to remove at least one gram of bone. Samples were sent to the W.M. Keck Carbon Cycle AMS Laboratory, University of California, Irvine (UCIAMS) for AMS radiocarbon dating.

### AMS methods

At UCIAMS, bone collagen was extracted and purified using the modified Longin method with ultrafiltration [50, 51]. Samples (200–400 mg) were demineralized for 24−36 h in 0.5 N HCl at 5°C, followed by a brief (< 1 h) alkali bath in 0.1 N NaOH at room temperature to remove humates. The pseudomorph was rinsed to neutrality in multiple changes of 18.2 MΩ H_2_O, and then gelatinized for 10 h at 60°C in 0.01 N HCl. Gelatin solution was pipetted into precleaned Centriprep® 30 ultrafilters (retaining >30 kDa molecular weight gelatin) and centrifuged three times for 20 min, diluted with 18.2 MΩ H_2_O, and centrifuged three more times for 20 min to desalt the solution. More detailed ultrafilter cleaning methods are described by McClure and colleagues [52]. Ultrafiltered collagen was lyophilized and weighed to determine the percent yield as a first evaluation of the degree of bone collagen preservation. All δ^13^C and δ^15^N values were measured to a precision of <0.1‰ and <0.2‰, respectively, on aliquots of ultrafiltered collagen, using a Fisons NA1500NC elemental analyzer/Finnigan Delta Plus isotope ratio mass spectrometer. Sample quality was evaluated by % crude gelatin yield, %C, %N, and C:N ratios before AMS radiocarbon dating. C:N ratios for the samples ranged from 3.2 to 3.4, indicating good collagen preservation and within the threshold advocated for by DeNiro (2.9−3.6) and van Klinken (3.1−3.5) [53, 54]. Given the initial collagen yield of 0.9% for UCIAMS 229652, the sample was reanalyzed as sample UCIAMS 229653 with a collagen yield of 2.0%. However, both dates are included in this study. Radiocarbon samples (~2.5 mg) were combusted for 3 hours at 900°C in vacuum sealed quartz tubes with CuO wire and Ag wire. Sample CO_2_ was reduced to graphite at 550°C using H2 and a Fe catalyst, with reaction water drawn off with Mg(ClO_4_)_2_ [55]. Graphite samples were pressed into targets in Al cathodes and loaded on the target wheel for AMS analysis. Radiocarbon ages were corrected for mass-dependent fractionation with measured δ13C values on the AMS [28] and compared with samples of ^14^C free whale bone and mammoth bone.

### Bayesian statistical modeling

The construction and modeling of archaeological chronologies through Bayesian approaches incorporates prior information about the archaeological site(s) and regional cultural histories, emphasizing the context, provenience, relative dating, and stratigraphic relationships of samples [13, 27, 38, 56, 57]. Given that the primary goal in the current research is to provide a reliable and precise chronological model for the occupation of the rectangular plank house structure and the Palmrose site generally, half the samples in the present study are derived from excavation units within the plank house feature. The remaining samples derive from north of the house feature in sediments interpreted as stratigraphically intact by Phebus and Drucker [40, 41]. Therefore, the prior knowledge used in chronological models’ construction includes archaeological context, stratigraphic, and sedimentary data derived from archival field notes and previously published reports [40–42].

In this analysis, radiocarbon dates were calibrated using the IntCal20 Northern Hemisphere calibration curve and Bayesian models developed and tested in OxCal 4.4 [46, 47]. Bayesian modeling allows researchers to statistically test potential chronological events providing probabilities for *terminus post quem*, *terminus ante quem*, chronological sequence, phase(s), and their chronological span [46, 56]. As noted by Bronk Ramsey [46], a vital consideration of any chronological model is the recognition that stratigraphic information may not necessarily reflect chronological order; therefore, individual agreement indices and three other indices, model agreement, overall agreement, and convergence, are crucial.

OxCal chronological modeling calculates an individual agreement (A) index for each dated item or sample and an index for the model (*A*_*model*_), which is a measure of the agreement between the model and the observed data [46]. An overall agreement (*A*_*overall*_) index for the model is also determined, calculated from the individual agreement indices [46, 56]. Individual sample indices, model indices, and overall indices can have a 100% value but can be higher and might fall as low as 60% to 0%. As Bronk Ramsey [46] notes, model agreement indices should not fall below 60%. If the model agreement index falls below 60% (analogous to 0.05 significance level in a *Χ*^2^ test), the radiocarbon results or the models are problematic [56]. Therefore, these various agreement indices allow researchers to test unreliable models, dates, or identify intrusive dates [46, 56].

The combination of Bayesian analysis and chronological modeling of 12 new AMS radiocarbon dates for the Palmrose site and the plank house feature, along with field notes and provenience information, provides an excellent opportunity to create a revised and precise chronology for the Palmrose site. These new data have the potential to change our understanding of site chronology, the development of semi- to fully-sedentary lifeways on the northern Oregon Coast, and alter regional chronologies broadly [6, 9].

In the models’ construction, I assumed that all deposits were in undisturbed stratigraphic order, based on information in the existing field notes. To test this assumption and the stratigraphic integrity of the site and radiocarbon samples, I initially created simple calibration models and sequences for individual excavation units, applying priors from stratigraphic levels within each unit, before constructing more intricate chronological modeling following Sanchez and colleagues [9]. Radiocarbon dates were placed in a sequence in OxCal with boundary start and end dates calculated.

## Results

Eleven of the 12 samples produced sufficient collagen yield. However, the analysis of sample 1593–9 from unit SE5F level 5 resulted in zero collagen yield and was not processed further. As previously noted, sample 1593–4 has duplicate dates resulting in 12 new AMS dates for the site. The conventional radiocarbon ages for the 11 samples range from 2135 ± 20 to 1785 ± 20 (Table 2). Based on unmodeled calibration for the 12 dates, the site may have been inhabited from 345−55 cal BC to cal AD 225−340. To test the general stratigraphic integrity of the samples, I calibrated each unit through Bayesian methods. I created sequences for each unit by organizing samples based on excavation levels and included start and end boundaries to test for and identify radiocarbon reversals before merging all dates in a broader chronological model integrating additional stratigraphic data. All radiocarbon ranges presented below represent 95.4% probability.

**Table 2:** Conventional and calibrated AMS ^14^C dates on cervid bone from the Palmrose site. Context designations derived from Phebus and Drucker field notes.

Sample ID	Taxon	Context	Element	UCIAMS #	δ^13^C (‰, VPDB)	δ^15^N (‰, Atm N2)	C/N	Provenience(Unit-Level)	Conventional ^14^C Age BP	cal BC/AD (95.4% CI)
1593–1	*Odocoileus sp*.	Basal ash lens/crushed shell above the subsoil	Calcaneus	229649	-22.6	2.5	3.2	NE4B- 8	2135 ± 20	345−55 BC
1593–2	*Cervus elaphus*	Terminal crushed shell and humus deposits	Phalanx	229650	-21.1	2.9	3.3	NE4B-2	1845 ± 20	AD 125−240
1593–3	*Odocoileus sp*.	Intermediate deposits with crushed shell, humus, rock	Astragalus	229651	-24.2	3.1	3.2	NE4B-6	1930 ± 20	AD 25−205
1593–4	*Cervus elaphus*	Terminal crushed shell and humus deposits	Astragalus	229652	-21.7	3.9	3.3	NE1K-2	1810 ± 20	AD 205−330
1593–4 (Dup.)	*Cervus elaphus*	Terminal crushed shell and humus deposits	Astragalus	229653	-21.7	4.2	3.4	NE1K-2	1785 ± 20	AD 225−340
1593–5	*Cervus elaphus*	Intermediate crushed shell and humus deposits	Lower/Upper Premolar	229654	-21.2	5.2	3.2	NE1K-4	2125 ± 20	340−50 BC
1593–6	*Cervus elaphus*	Lower crushed shell deposits	Astragalus	229655	-25.1	3.7	3.3	NE1K-6	2100 ± 20	175−45 BC
1593–7	*Odocoileus sp*.	Basal ashy, crushed shell deposits	Astragalus	229656	-25.0	2.2	3.3	SE5F-10	2095 ± 20	170−45 BC
1593–8	*Cervus elaphus*	Terminal shell and humus deposits	Astragalus	229657	-21.3	3.2	3.3	SE5F-7	1815 ± 20	AD 170−330
1593–9	*Cervus elaphus*	Surface deposits	Astragalus	---	-25.3	3.6	---	SE5F-5	---	---
1593–10	*Cervus elaphus*	Lower crushed shell deposits	Calcaneus	229658	-24.9	4.2	3.3	SE3C-9	2035 ± 20	100 BC−AD 55
1593–11	*Cervus elaphus*	Ashy sand/crushed shell deposits	Astragalus	229659	-22.0	3.5	3.2	SE3C-7	1885 ± 20	AD 80−220
1593–12	*Odocoileus sp*.	Upper crushed shell and humus deposits	Astragalus	229660	-22.6	2.5	3.3	SE3C-3	1840 ± 20	AD 125−245

### Chronology building first iteration: Excavation unit stratigraphic models

#### NE1K

**Fig 3:**
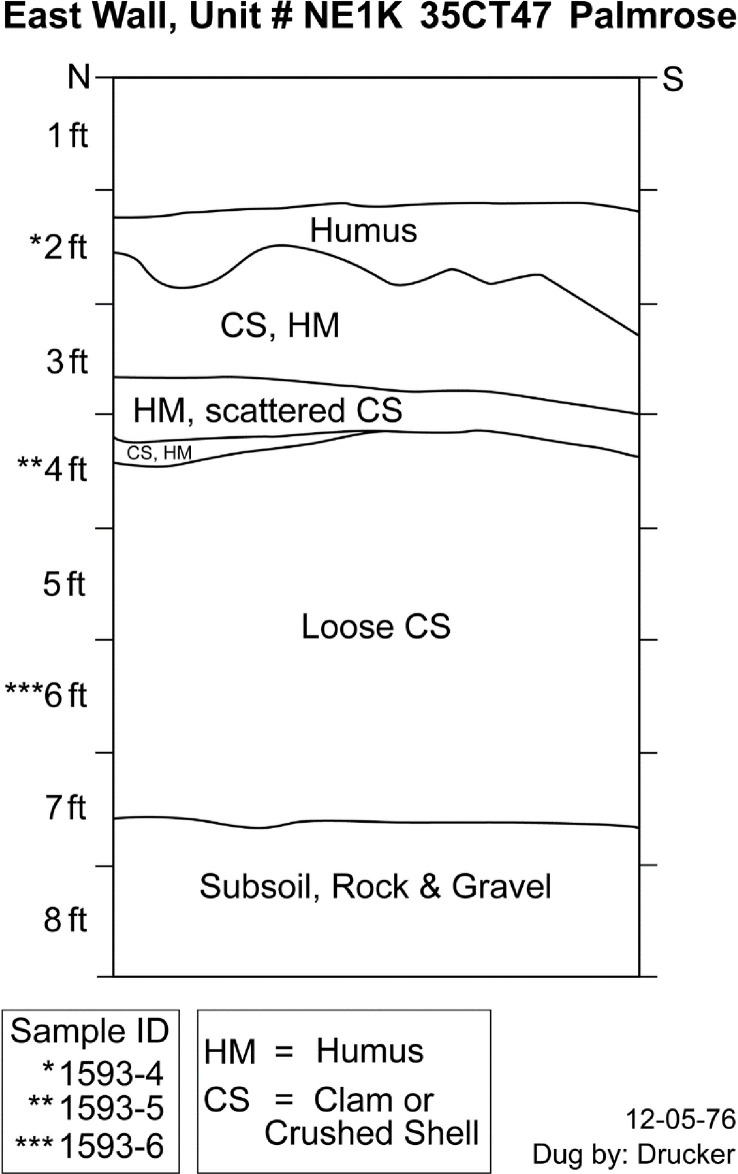
Palmrose unit NE1K east wall profile. Level provenience of radiocarbon samples noted. Adapted from Palmrose excavation notes.

The cervid remains from excavation unit NE1K, outside of the plank house structure, include three samples from levels 6, 4, and 2 (Fig 3). Level 6 appears to represent shell midden deposits that overlie basal components of the occupation. Level 4 is an intermediate level that crosscuts three stratigraphic differences, including shell midden, crushed shell, and humus deposits. Level 2 represents terminal shell midden deposits overlaid by humus.

The conventional radiocarbon age for the specimens ranges from 2100 ± 20 BP to 1785 ± 20. In this modeling stage, samples from level 2, including two duplicates dates from the same sample, are combined using the R_Combine command. The agreement indices for the model are *A*_*model*_ = 90.6 and *A*_*overall*_ = 91.7 within the tolerance suggested by Bronk Ramsey [46]. No statistically significant stratigraphic reversals are present in the unit. The units modeled sequence suggests a possible start of occupation between *1125−65 cal BC* and ending around *cal AD 220−1145* with modeled radiocarbon dates from the basal and upper components of the midden spanning *180−60 cal BC* to *cal AD 215−325*, Table 3.

**Table 3:** Radiocarbon dates for unit NE1K, including modeled sequence, 95% probability ranges, and boundaries.

UCIAMS #	Sample ID	Level	Conv. ^14^C age (BP)	*Modeled 95*.*4% CI (BC/AD)*
**Boundary**		*End of occupation*	---	*AD 220*−*1145*
**229653**	1593–4	2 (Dup.)	1785 ± 20	---
**229652**	1593–4	2	1810 ± 20	---
**R_Combine**	1593–4	2	---	*AD 215*−*325*
**229654**	1593–5	4	2125 ± 20	*155*−*50 BC*
**229655**	1593–6	6	2100 ± 20	*180*−*60 BC*
**Boundary**		*Start of occupation*	---	*1125*−*65 BC*

#### NE4B

**Fig 4:**
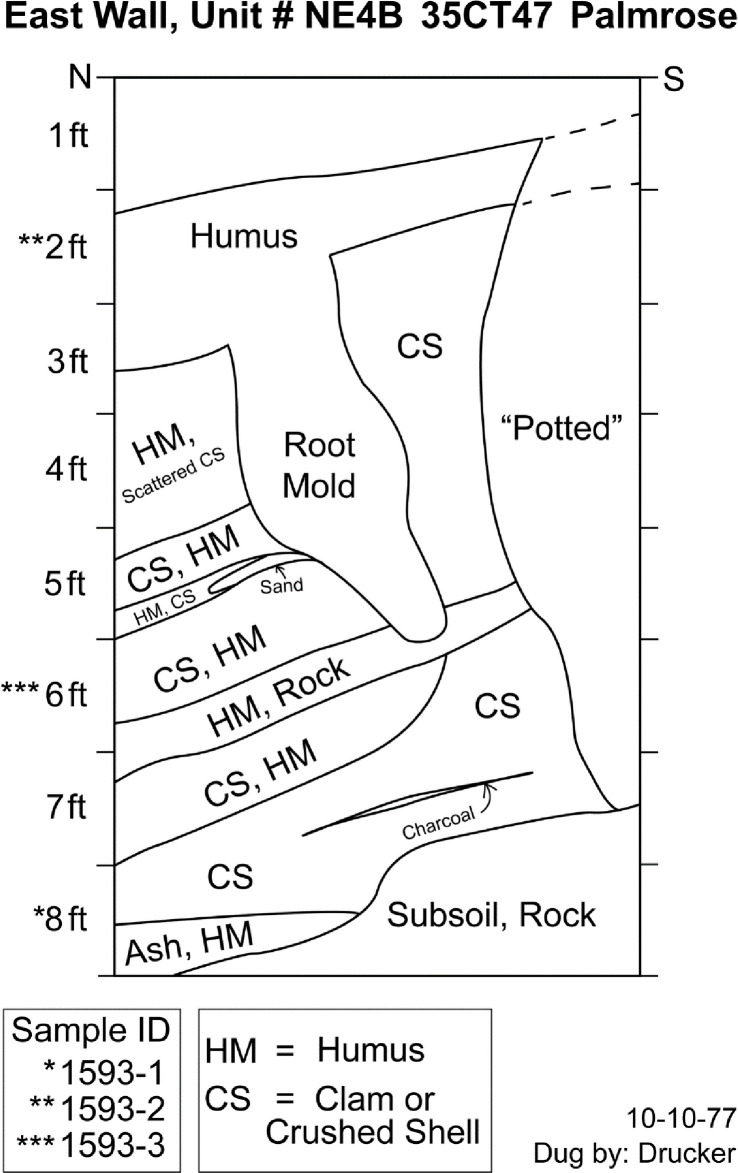
Palmrose unit NE4B east wall profile. Level provenience of radiocarbon samples noted.

Radiocarbon samples from excavation unit NE4B are not within the plank house structure. The unit was partially disturbed by looters on its southern portion. Three samples from the unit were selected for analysis from levels 8, 6, and 2 (Fig 4). Level 8 represents midden deposits near the basal component of the occupation, which crosscuts at least two stratigraphic differences noted by the field crew, including ashy deposits and shell midden, above the previously noted rocky subsoil. Level 6 crosscuts several stratigraphic differences within the unit, including crushed shell midden, humus, and rock deposits. Lastly, level 2 represents terminal shell midden deposits overlain by humus.

The conventional radiocarbon ages for the specimens range from 2135 ± 20 to 1845 ± 20. The agreement indices for the model are *A*_*model*_ = 94.9 and *A*_*overall*_ = 95.6 within the tolerance suggested by Bronk Ramsey [46]. No statistically significant stratigraphic reversals are present in the unit. The units modeled sequence suggests a possible start of occupation at *1130−55 cal BC* and ending around *cal AD 130−1105* with modeled radiocarbon dates from the basal and upper components of the midden spanning *340−50 cal BC* to *cal AD 125−240*, Table 4.

**Table 4:** Radiocarbon dates for unit NE4B, including modeled sequence, 95% probability ranges, and boundaries.

UCIAMS #	Sample ID	Level	Conv. ^14^C age (BP)	*Modeled 95*.*4% CI (BC/AD)*
**Boundary**		*End of occupation*	---	*AD 130*−*1105*
229650	1593–2	2	1845 ± 20	*AD 125*−*240*
229651	1593–3	6	1930 ± 20	*AD 20*−*165*
229649	1593–1	8	2135 ± 20	*340*−*50 BC*
**Boundary**		*Start of occupation*	---	*1130*−*55 BC*

#### SE3C

**Fig 5:**
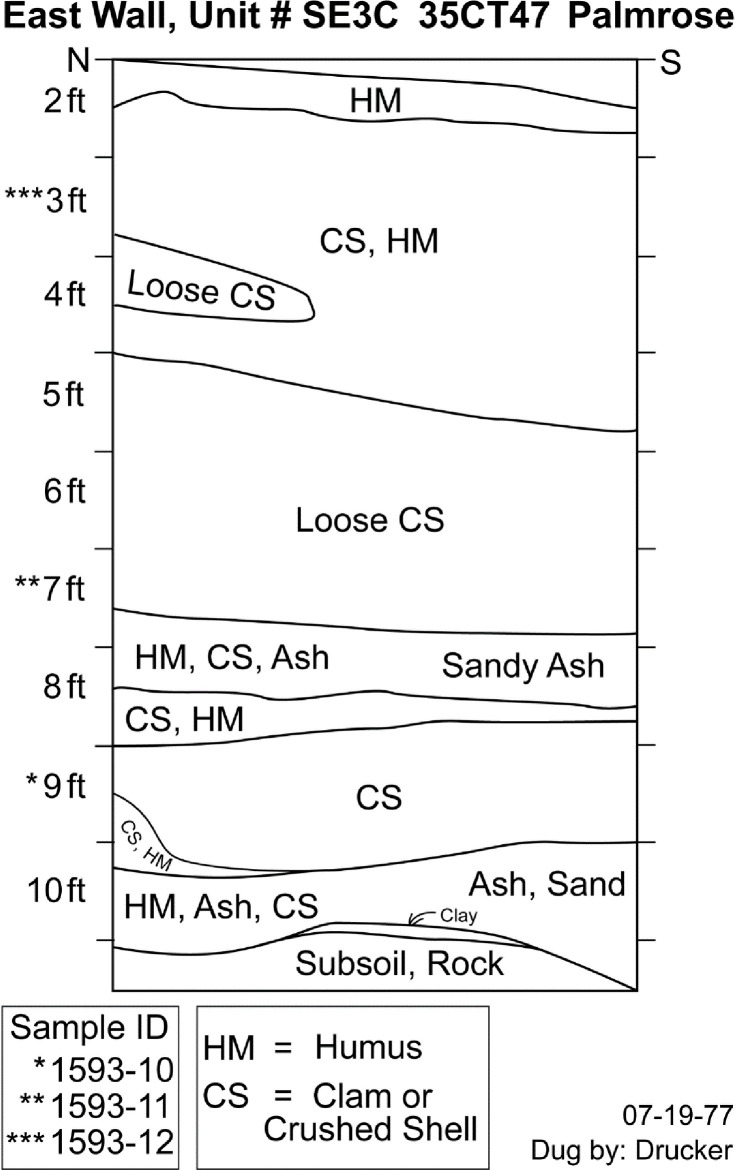
Palmrose unit SE3C east wall profile. Level provenience of radiocarbon samples noted.

Samples from excavation unit SE3C are derived from deposits associated with and in the plank house feature, which offers the potential to approximate the extent of plank house occupation, potential rebuilding episodes, and site occupation. Three samples from the unit were selected for analysis from levels 9, 7, and 3 (Fig 5). Level 9 represents shell midden deposits directly above the ashy sand stratigraphy identified by Phebus and Drucker and by OSMA archaeologists, which has been interpreted as the initial house building episode. Level 7 crosscuts a second ashy sand deposit and a shell midden deposit that overlies the basal ashy sand. Level 3 appears to represent shell midden deposits near the termination of the midden formation and occupation.

The conventional radiocarbon ages for the specimens range from 2035 ± 20 to 1840 ± 20. The agreement indices for the model are *A*_*model*_ = 102 and *A*_*overall*_ = 101.3 within the tolerance suggested by Bronk Ramsey [46]. No statistically significant stratigraphic reversals are present in the unit. The modeled sequence suggests a possible start of occupation around *1060 cal BC−cal AD 60* and ending around *cal AD 130−1070* with modeled radiocarbon dates from the middens basal and upper components of the midden spanning *95 cal BC−cal AD 60* to *cal AD 130−245*, Table 5.

**Table 5:** Radiocarbon dates for unit SE3C, including modeled sequence, 95% probability ranges, and boundaries.

UCIAMS #	Sample ID	Level	Conv. ^14^C age (BP)	*Modeled 95*.*4% CI (BC/AD)*
**Boundary**		*End of occupation*	---	*AD 130*−*1070*
229660	1593–12	3	1840 ± 20	*AD 130*−*245*
229659	1593–11	7	1885 ± 20	*AD 80*−*215*
229658	1593–10	9	2035 ± 20	*95 BC*−*AD 60*
**Boundary**		*Start of occupation*	---	*1060 BC*−*AD 60*

#### SE5F

**Fig 6:**
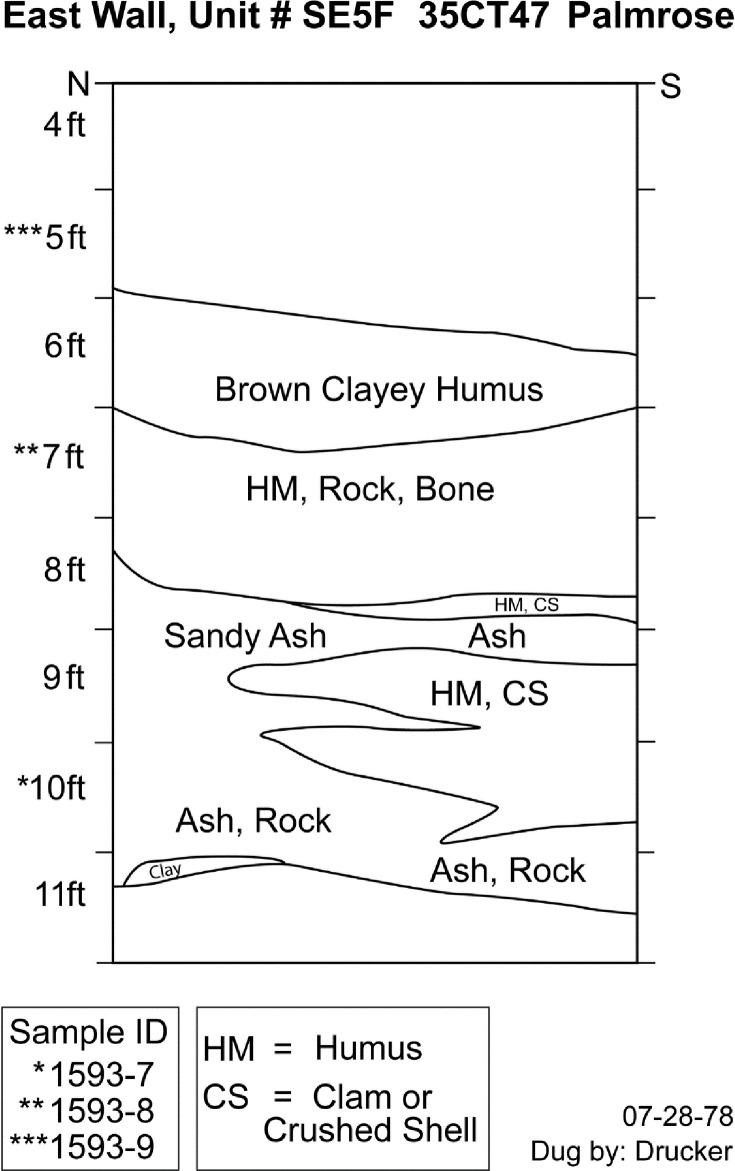
Palmrose unit SE5F east wall profile. Level provenience of radiocarbon samples noted.

Lastly, unit SE5F is also within the plank house structure. Three samples were submitted for radiocarbon dating from levels 10, 7, and 5 (Fig 6). However, sample 1593–12 from level 5 lacked sufficient collagen preservation for radiocarbon dating—level 5 represented surface materials from the midden. Level 10 appears to be associated with ashy shell midden deposits above the subsoil, potentially indicative of initial site occupation. Level 7 represents terminal midden deposits above the middle ashy sand deposit found in SE3C, demonstrating consistency between the house occupation’s midden deposits.

The conventional radiocarbon ages for the specimens range from 2095 ± 20 to 1815 ± 20. The agreement indices for the model are *A*_*model*_ = 100.4 and *A*_*overall*_ = 100.4 within the tolerance suggested by Bronk Ramsey [46]. No statistically significant stratigraphic reversals are present in the unit. The units modeled sequence based on the available data suggests a possible start of occupation at *1150−55 cal BC* and ending around *cal AD 210−1200* with modeled radiocarbon dates from the middens basal and upper components spanning *170 cal BC−cal AD 55* to *cal AD 130−325*, Table 6.

**Table 6:** Radiocarbon dates for unit SE5F, including modeled sequence, 95% probability ranges, and boundaries.

UCIAMS #	Sample ID	Level	Conv. ^14^C age (BP)	*Modeled 95*.*4% CI (BC/AD)*
**Boundary**		*End of occupation*	---	*AD 210−1200*
---	1593–9	5	---	---
229657	1593–8	7	1815 ± 20	*AD 130−325*
229656	1593–7	10	2095 ± 20	*170 BC−AD 55*
**Boundary**		*Start of occupation*	---	*1150−55 BC*

### Chronology building second iteration: Palmrose occupation sequence

**Fig 7:**
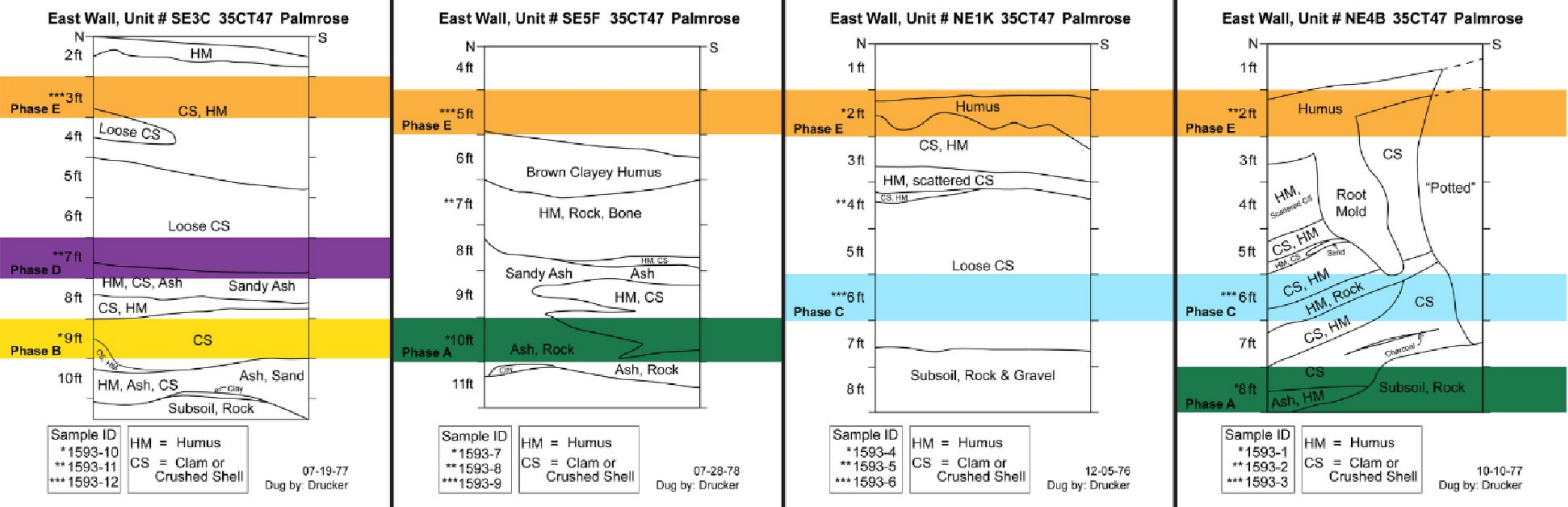
Unit profiles with phase designations derived from stratigraphic data from archived Phebus and Drucker field notes.

Based on the overall agreement between the sequences from the individual units, I constructed a chronological sequence model for the Palmrose site by analyzing the individual unit profiles for the four units. Overall, while there are differences across the four units, a general trend occurs across all four, which informed initial sample selection and the model’s construction. Each unit’s basal component includes sand or ashy sand deposits overlain by shell midden, except for unit NE1K. Therefore, the first boundary I included in the model was the ashy sand/ash and rock lens, which I termed Phase A (Fig 7). These deposits include level 8 from NE4B and level 10 from SE5F, both within the house feature and interpreted as indicative of the house’s initial occupation. Next, I termed level 9 from unit SE3C as Phase B as the level generally corresponds with and includes shell midden that does not contain components of the ashy sand level, which it overlies. These stratigraphic components of the profiles correspond with shell midden deposits often noted as loose midden or crushed shell midden in the field notes.

Phase C comprises stratigraphic level 6 in units NE1K and NE4B, both of which appear to represent midden deposits above the basal deposits or the ashy sand but not associated with the intermediate sand lens, especially the small sand lens present in NE4B. Phase D is based on a single date from SE3C level 7, which crosscuts the intermediate sand lens’s upper deposits and the overlying shell midden. I interpret these deposits to represent the second house construction episode or, at a minimum, a reestablishment of the house floor through the addition of new sand. Lastly, Phase E represents shell midden overlying the second intermediate sand lens until midden formation ends.

**Fig 8:**
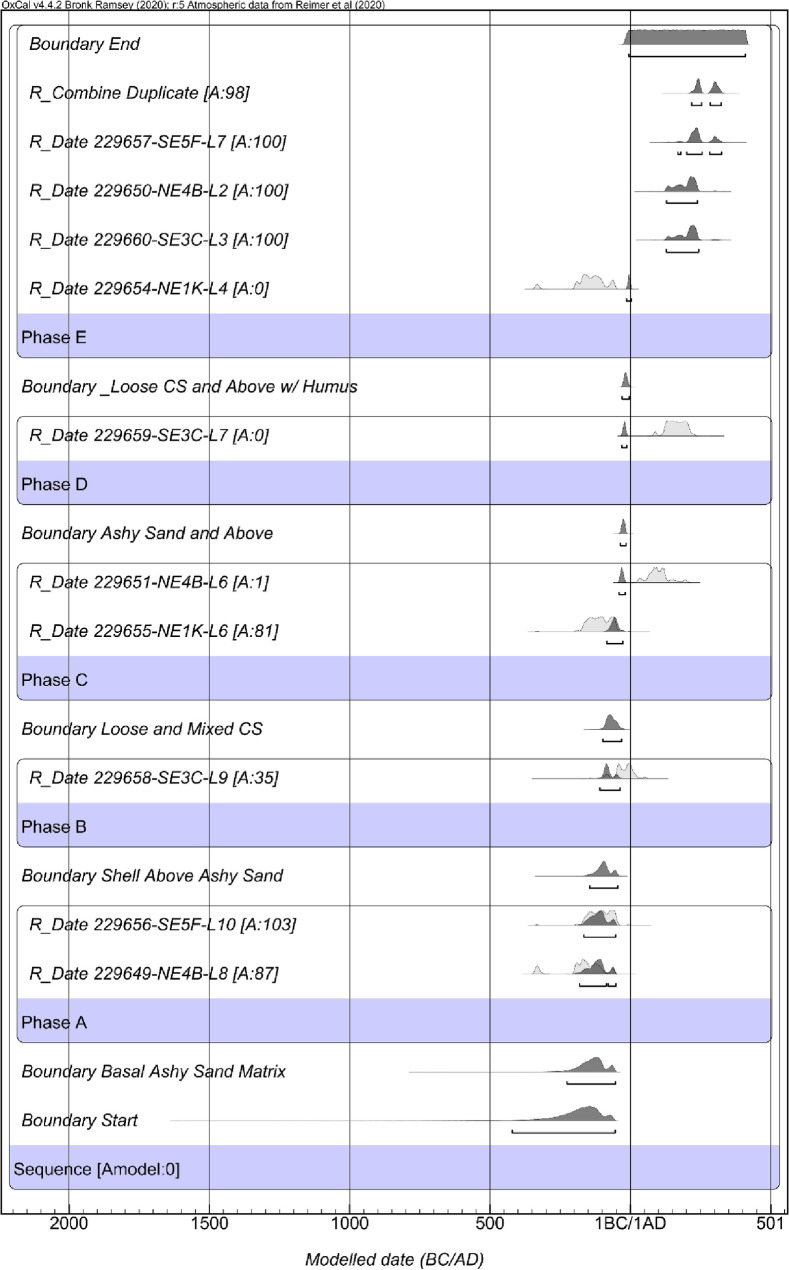
Results of the second iteration of the Palmrose chronological modeling.

The second iteration of chronological models informed by the stratigraphic variation fails due to two stratigraphic reversals within the model resulting in a model agreement index of *A*_*model*_ = 0 (Fig 8). Therefore, the second iteration model results informed the treatment of samples in the creation of the third iteration of modeling. Specifically, I excluded two significant outliers found in model two. Both outliers in the model derive from NE1K levels 6 and 4. While the exact cause of the stratigraphic reversals is unknown, given the complex stratigraphy for the site, evidence for multiple rebuilding episodes, and significant looting, it is not surprising to discover discontinuities in the site stratigraphy and radiocarbon reversals. As noted by Bayliss and colleagues [56] and Bronk Ramsey [46], such findings from model construction are critical a priori information that can and should be used in later iterations of model building.

### Chronology building third iteration: Palmrose occupation sequence excluding outliers

**Fig 9:**
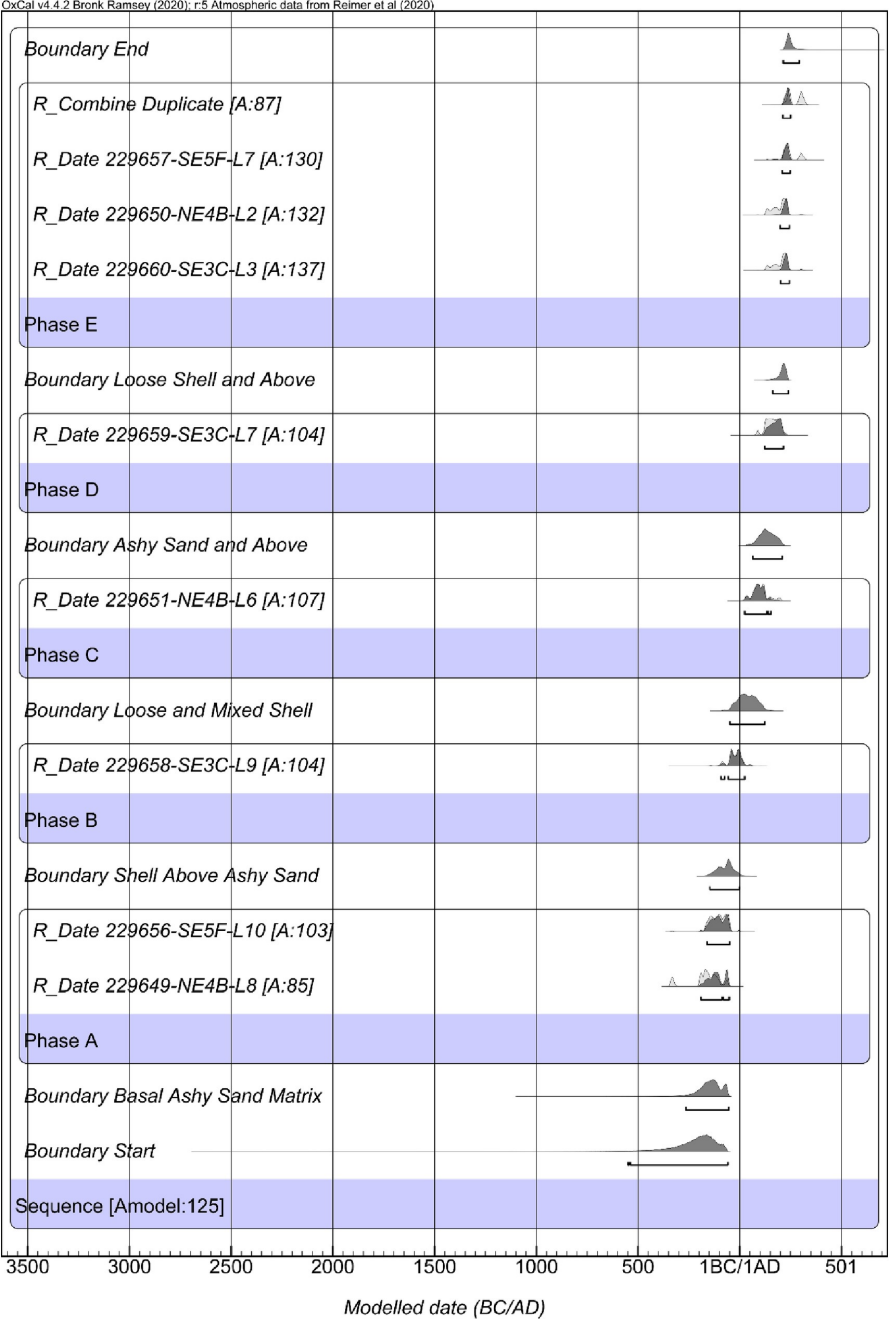
Results of the third iteration of the Palmrose chronological modeling.

With the exclusion of the outliers from NE1K, the constructed Bayesian model of the Palmrose sequence based on my interpretation of the stratigraphy of the unit profiles suggests that the site and plank house’s primary occupation may have spanned from *580−55 cal BC* to cal *AD 210−300*, based on modeled start and end calculations in the sequence (Fig 9 and Table 7). The agreement indices for the model are *A*_*model*_ = 124.9 and *A*_*overall*_ = 127.6. Based on the two radiocarbon assays—level 8 from NE4B and level 10 from SE5F, both within the house feature—Phase A, the basal sandy ash lens overlying the subsoil may have been occupied from *195−50 cal BC* (Figs 7 and 9).

**Table 7:** The third iteration of the Palmrose chronological modeling, including modeled sequence, 95% probability ranges, and boundaries. Model indices: *Amodel* = 124.9 and *Aoverall* = 127.6.

Name	*Modeled 95*.*4% CI (BC/AD)*	Agreement	Convergence
Difference Span	*855 to 290 cal yr*		97.5
Boundary End	*AD 210−300*		99.5
*R_Combine Duplicate*	*AD 210−255*	87	99.7
*R_Date 229657-SE5F-L7*	*AD 205−250*	129.7	99.8
*R_Date 229650-NE4B-L2*	*AD 195−245*	132.3	99.8
*R_Date 229660-SE3C-L3*	*AD 200−245*	137.1	99.7
Phase E			
Boundary Loose Shell and Above	*AD 160−240*		99.6
*R_Date 229659-SE3C-L7*	*AD 120−215*	104.4	100
Phase D			
Boundary Ashy Sand and Above	*AD 65−210*		99.9
*R_Date 229651-NE4B-L6*	*AD 25−155*	106.7	99.9
Phase C			
Boundary Loose and Mixed Shell	*50 BC−AD 125*		99.9
*R_Date 229658-SE3C-L9*	*95 BC−AD 25*	104	99.9
Phase B			
Boundary Shell Above Ashy Sand	*150−1 BC*		99.9
*R_Date 229656-SE5F-L10*	*165−50 BC*	103.3	99.8
*R_Date 229649-NE4B-L8*	*195−50 BC*	84.8	99.7
Phase A			
Boundary Basal Ashy Sand Matrix	*270−50 BC*		99.5
Boundary Start	*580−55 BC*		97
Sequence			

Phase B spans from *95 cal BC−cal AD 25* and is derived from a single date from unit SE3C level 9. It represents shell midden that does not contain components of the basal ashy sand level that it overlies or the sand and ash lens above. Phase C is represented by a single date from NE4B level 6 and spans from *cal AD 25−cal AD 155* and appears to represent midden deposits above the basal ashy sand but not associated with the intermediate sand lens, especially the small sand lens present in NE4B.

Phase D spans from *cal AD 120−215* and is derived from a single date from unit SE3C level 7. Based on stratigraphic data, this sample crosscuts the intermediate sand lens’s upper deposits and the overlying shell midden. I interpret these deposits to represent the second house construction episode or, at a minimum, a reestablishment of the house floor through the addition of new sand.

Lastly, Phase E represents shell midden above the intermediate sand lens and spans from *cal AD 200−255*. These data are derived from five radiocarbon dates from SE3C level 3, NE4B level 2, SE5F level 7, and NE1K level 2. Therefore, the Bayesian models I constructed in this study based on my interpretations of the site stratigraphy, previous interpretations of the site, and the Bayesian modeling results may indicate three occupation phases and two house rebuilding episodes. The first occupation occurred sometime between *195 cal BC−cal AD 25* during Phase A and B. The second building episode spanned *cal AD 25−215* sometime between Phase C and D. The terminal occupation occurred sometime between *cal AD 200−255* during Phase E.

### Chronology building fourth iteration: Palmrose house occupation sequence

As previously mentioned, one of the primary goals of the present study is to define the duration of the Palmrose plank house occupation. Given that two excavation units directly correlate to the house feature, I created a fourth chronological model using these units solely. Like the third chronological model, the fourth model relies heavily on the excavation unit profiles and stratigraphic data reported by Phebus and Drucker [40, 41]. For example, the basal component of unit SE3C level 10 and 9 and unit SE5F level 11 and 10 include ashy, sandy, rock, clay deposits, and dense shell midden that overlie the rocky subsoil of the Palmrose site.

Unit SE3C levels 8 and 7 and SE5F levels 9 and 8 indicate a change in stratigraphy with a second intermediate ashy sand, crushed shell, and humus lens that I have interpreted as a second house occupation and reestablishment of an interior floor. Ancient floor zones often include ash and charcoal [58]. Hearths were often sand lined in Northwest Coast style plank houses [58]. Lastly, levels 6–2 in unit SE3C and levels ~7–6 in unit SE5F have been interpreted as indicative of the plank house’s terminal occupation. Based on these observances, the fourth chronological sequence may provide a refined model for the house occupation.

**Fig 10:**
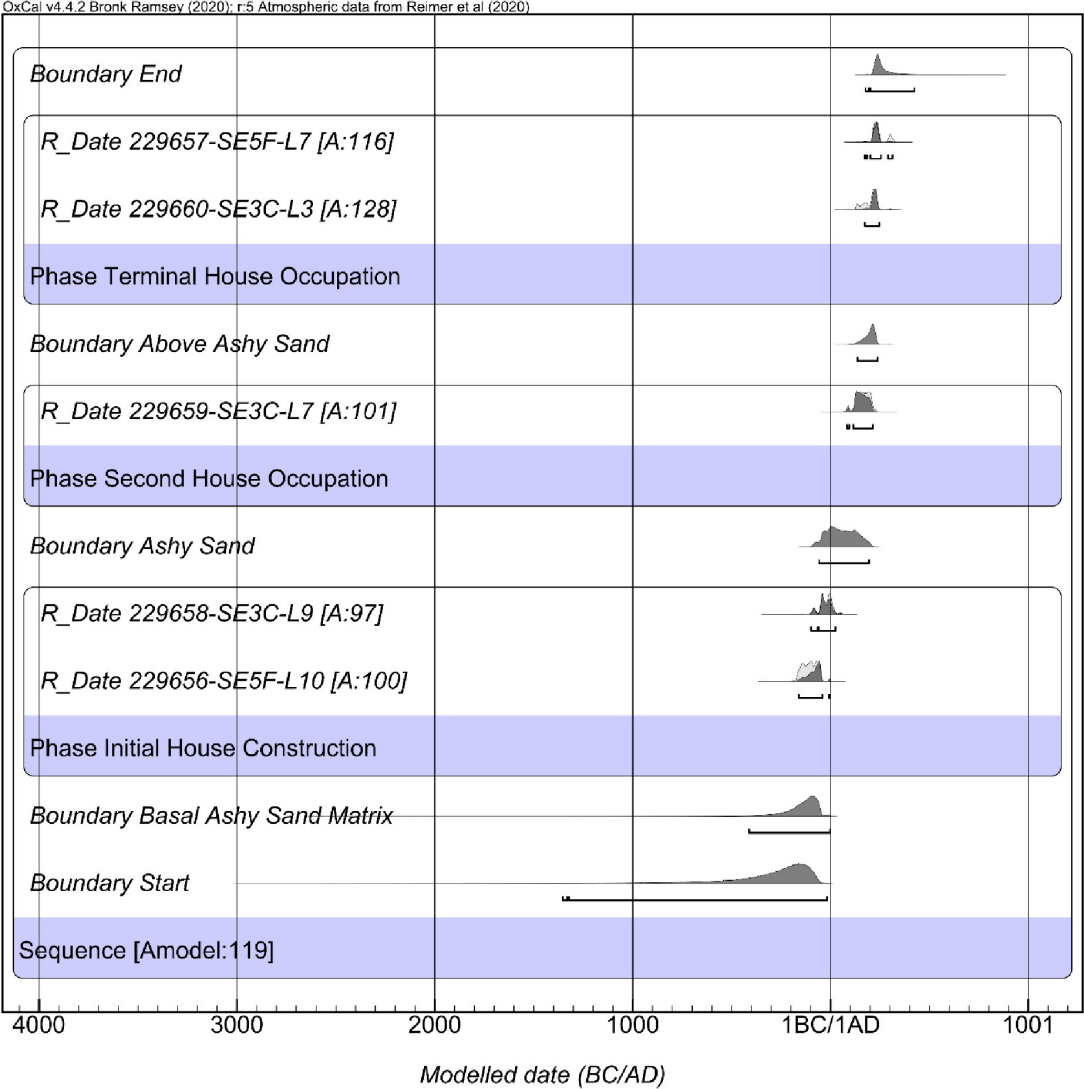
Results of the fourth iteration of the Palmrose chronological modeling.

The constructed Bayesian model results for the sequence suggest the maximum range of the plank house occupation may have spanned from *1360−10 cal BC* to *cal AD 170−430*, based on modeled start and end calculations in the sequence (Table 8, Fig 10). The agreement indices for the model are *A*_*model*_ = 119.4 and *A*_*overall*_ = 118.4.

**Table 8:** The fourth iteration of the Palmrose chronological modeling, including modeled sequence, 95% probability ranges, and boundaries. Model indices: *Amodel* = 119.4 and *Aoverall* = 118.4.

Name	*Modeled 95*.*4% CI (BC/AD)*	Agreement	Convergence
Difference Span	*1670 to 250 cal yr*		97.9
Boundary End	*AD 170−430*		99.4
*R_Date 229657-SE5F-L7*	*AD 170−320*	115.9	99.8
*R_Date 229660-SE3C-L3*	*AD 170−250*	128.1	99.9
Phase Terminal House Occupation			
Boundary Above Ashy Sand	*AD 130−240*		99.9
*R_Date 229659-SE3C-L7*	*AD 80−220*	101.1	99.9
Phase Second House Occupation			
Boundary Ashy Sand	*60 BC−AD 200*		99.7
*R_Date 229658-SE3C-L9*	*100 BC−AD 30*	96.9	99.9
*R_Date 229656-SE5F-L10*	*160−1 BC*	100.2	99.8
Phase Initial House Construction			
Boundary Basal Ashy Sand Matrix	*420−10 BC*		99.6
Boundary Start	*1360−10 BC*		98.4
Sequence			

**Fig 11:**
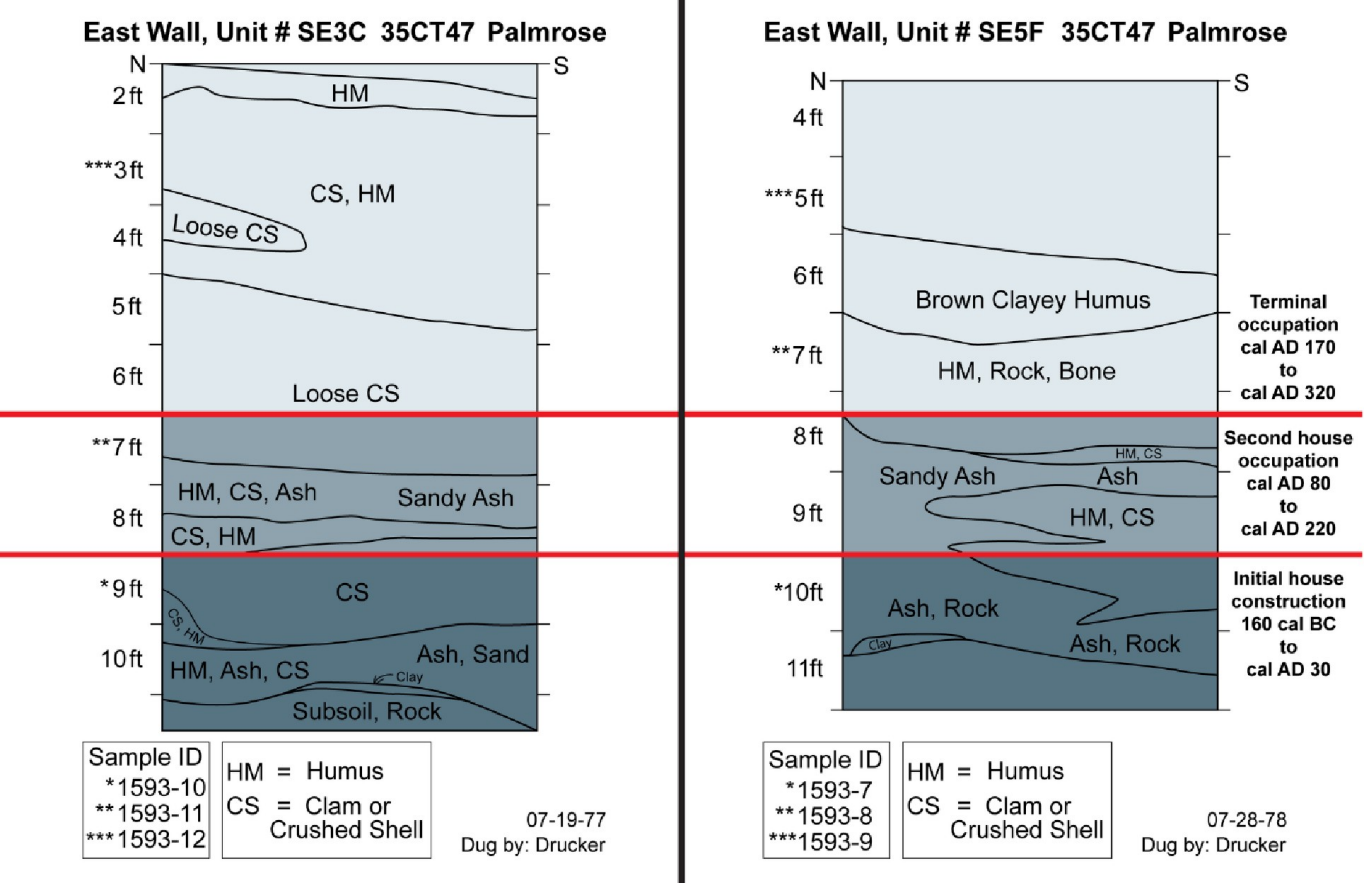
Unit SE3C and SE5F profiles with house occupation designations derived from reported stratigraphic data by Phebus and Drucker (40, 41).

The Bayesian modeling suggests that three plank house occupation periods may have occurred. The first suggests an occupation from *cal BC 160−cal AD 30* and is associated with midden deposits above the subsoil, including ashy sand, rock, clay, and shell midden in units SE3C and SE5F (Fig 11). The second occupation represented by a single date from unit SE3C and derived from the second intermediate sand lens or the shell midden overlying the sand deposit suggests that the second occupation period may have spanned from *cal AD 80− cal AD 220* (Fig 11). Lastly, based on two radiocarbon dates—one from SE5F level 7 that overlies the sand ash lens and another date from SE3C level 3—the plank house’s terminal occupation likely spans from *cal AD 170−cal AD 320* (Fig 11).

## Discussion

Radiocarbon dating by Phebus and Drucker [40, 41] and Connolly [42] suggest that the Palmrose site was inhabited from 2340 cal BC to cal AD 640. Interpretations of the dates by Phebus and Drucker [40, 41] and Connolly [42] suggest that the plank house may have been inhabited in three episodes, with the earliest occurring from 700−600 cal BC, intermediate occupation from 300−200 cal BC, and the terminal occupation around cal AD 200−300.

In this study, the third iteration model suggests start and end boundaries from *580−55 cal BC* to *cal AD 210−300*. The fourth iteration model start and end boundaries range from *1360−10 cal BC* to *cal AD 170−430*. The third model iterations indicate the possibility of three occupation episodes, dated between *195 cal BC−cal AD 25*, *cal AD 25−215*, and *cal AD 200−255*. The fourth model suggests the three occupations of the house may have occurred from *cal BC 160−cal AD 30*, *cal AD 80−cal AD 220*, and *cal AD 170 −cal AD 320*. These results are in sharp contrast to previous reports. The reasons for that are that the models presented in this study would constrain the maximum range of the site and plank house occupation to *1360−10 cal BC* to *cal AD 170−430* (fourth model iteration), based on modeled start and end calculations, rather than 2340 cal BC to cal AD 640 as suggested by Phebus and Drucker [40, 41] and Connolly [42]. The models in this study indicate that the three occupations of the plank house likely occurred in a much-constrained period and likely indicate a continuous occupation of the site. These new data affect regional chronologies and interpretations of human subsistence, occupation, and human-animal relationships across time and space discussed further below.

### Reconsidering the Seaside regional chronology: Implications for human-environmental relationships and subsistence practices

The long-standing regional chronology for the Seaside area was primarily comprised of radiocarbon dates from Palmrose, Avenue Q, and Par-Tee, with the majority of radiocarbon assays derived from Phebus and Drucker’s work. These data suggested the Palmrose site was inhabited from 2340 cal BC to cal AD 640, Avenue Q from 1925 cal BC to cal AD 995, and Par-Tee from 350 cal BC to cal AD 1150 [40–42]. Therefore, the previous Seaside regional chronology suggested that the Seaside area’s initial occupation began with the Palmrose occupation, followed by Avenue Q. It was long thought that Palmrose and Avenue Q were both occupied contemporaneously. Lastly, it was believed that the initial occupation of Par-Tee overlapped for a limited time with Palmrose and a more extended period with Avenue Q.

The Palmrose site economy has been interpreted as more terrestrially, marine, and riverine focused, while marine taxa dominate Par-Tee [30, 31]. These interpretations are derived from extensive faunal museum collections. For example, Colten [30, 31] suggests Palmrose has more bones of migratory marine mammals, such as northern fur seals (*Callorhinus ursinus*) and Steller sea lions (*Eumetopias jubata*), than Par-Tee. The Par-Tee marine mammal assemblage has many more bones of sea otters (*Enhydra lutris*) and harbor seals (*Phoca vitulina*) than Palmrose. In terms of birds, Par-Tee has many more pelagic bird species, such as albatross (Diomedeidae), shearwaters (*Puffinus* sp.), and murres (*Uria aalge*), than Palmrose. In contrast, Palmrose has the remains of more coastal and estuary birds, such as cormorants (Phalacrocoracidae), ducks and geese (Anatidae), and grebes (Podicipedidae) Colten [30, 31].

Sanchez and colleagues [59] recently conducted an ichthyofaunal analysis of the Par-Tee collection and compared their findings to previously reported data from the Palmrose and Avenue Q sites. As previously mentioned, the Palmrose faunal assemblage was recovered with 1/8 in. mesh sieves. The Palmrose site is dominated by salmon (*Oncorhynchus* sp.), representing 67% of the site assemblage. Therefore, it appears that the fishery’s focus was directed toward the acquisition of salmon supplemented by other fishes. Avenue Q was also recovered with 1/8 in. mesh sieves, with the fishery divided across multiple species including greenlings (Hexagrammidae), surfperches (Embiotocidae), skates (Rajidae), and hakes (Merlucciidae), among others, and suggest more variability and diversity in fishing practices, as no single fish organism dominates the assemblage as evidenced at Palmrose. Therefore, the Avenue Q fishery likely represents a broad-based fishery.

At Par-Tee, Phebus and Drucker recovered the faunal assemblage using 1/4 in. mesh sieves. It appeared to be a broad-based hook and line fishery focused on large fishes such as sturgeon (*Acipenser* sp.) and large predatory fishes such as lingcod (*Ophiodon elongatus*), rockfish (*Sebastes* sp.), and cabezon (*Scorpaenichthys marmoratus*) with limited evidence for salmon fishing. The inclusion of the fauna from bulk sediment samples hint at the possibility that mass-capture techniques were practiced targeting herrings (Clupeidae), Pacific tomcod (*Microgadus proximus*), smelts (Osmeridae), and Northern anchovy (*Engraulis mordax*) [59].

Previous research regarding the potential for cetacean hunting at Par-Tee is also significant. Losey and Yang [34] suggested the possibility that opportunistic whaling for humpback whales (*Megaptera novaeangliae*) occurred at the site. Radiocarbon dating by Sanchez and colleagues [36] suggested that the potential whaling event occurred around cal AD 430−550. Analysis of the Par-Tee and Palmrose marine mammal assemblage by Colten [30, 31] suggested that cetacean remains differ between the sites. Both sites had significant numbers of harbor porpoises (*Phocoena phocoena*). The Palmrose site had the remains of many bottlenose dolphins (*Tursiops truncata*), while Par-Tee has larger cetacean bones, notably those of Minke whale (*Balaenoptera acutorostrata*) and humpback. Subsequent analysis of the larger cetaceans by Wellman and colleagues [35] suggests the use of stranded whales may have been more common than opportunistic whaling. Analysis of small cetaceans by Loiselle [32] suggests that Par-Tee residents were more frequently hunting rather than scavenging the small cetaceans, predominantly harbor porpoise, Dall’s porpoise (*Phocoenoides dalli*), bottlenose dolphin (*Tursiops truncatus*), and Pacific white-sided dolphin (*Lagenorhynchus obliquidens*).

Consideration of the variation between the Palmrose and Par-Tee assemblages and, to a lesser extent Avenue Q, reveal several interpretations to explain these differences. First, the variation may result from the chronological separation of the sites [30, 31]. Second, there is the possibility of environmental variation in the Seaside area due to the potential infilling of an ancient bay in the sites’ vicinity [60]. Third, a cultural explanation has been suggested offering the possibility that different ethnic or tribal groups were living in close proximity, possibly reflective of historical patterns of Tillamook and Clatsop indigenous communities residing in Seaside at the time of European colonization [31]. Fourth, the variation may be driven by economic differences between the sites given the presence of a plank house at Palmrose and the lack of unambiguous residential structures at Par-Tee, especially as plank houses have been interpreted as the primary economic production and storage centers [60–62]. Fifth, the potential for differences in seasonal occupations of the sites [60].

The recent radiocarbon dating of the Palmrose and Par-Tee sites offers insights into the feasibility of these various possibilities. First, the interpretation that temporal differences between the Palmrose and Par-Tee sites may explain these differences is unlikely based on the refined chronology. Rather than the Palmrose and Par-Tee site occupations ranging from 2340 cal BC to cal AD 640 and 350 cal BC to cal AD 1150, the new chronological models suggest the maximum extent of the Palmrose occupation occurred from *1360−10 cal BC* to *cal AD 170−430* (fourth iteration model), but could be as constrained as *580−55 cal BC* to *cal AD 210−300* (third iteration model). The occupation of Par-Tee ranged from *cal AD ~100−800*. Therefore, the chronological difference between the two sites changes significantly.

Regarding environmental variation between the two sites, the revised chronology suggests previous chronological research related to the timing of the infilling of an ancient bay near Seaside needs to be reconsidered [60]. As the radiocarbon dates reported by Connolly [60] and Phebus and Drucker [40, 41] provided the basis for the analysis of molluscan remains by Connolly [60] and the subsequent interpretations of shifts in estuarine shellfish to open coast species, the findings of Sanchez and colleagues [9] and the present study strongly suggest the presently reported timing of the bay infilling should be reconsidered and reinvestigated, due to the inclusion of radiocarbon samples which do not adhere to chronometric hygiene standards as applied in this study. However, this study’s findings suggest the timing of the bay infilling occurred much more recently than previously believed. The present study cannot offer further support or refute interpretations regarding potential ethnic, seasonal, or economic variation between the sites or the use of different habitats by site inhabitants. However, the faunal data summarized does suggest differences in economic activities between the Palmrose and Par-Tee sites.

## Conclusions

AMS radiocarbon dating and Bayesian modeling for the Palmrose and Par-Tee sites significantly alter site-specific and regional chronological models altering interpretations regarding human economic and environmental variation across space and time. The study suggests the Palmrose site was inhabited much more recently than previously believed and indicates the antiquity of fully- to semi-sedentary communities along the Oregon Coast needs to be reconsidered. In addition, the revised Palmrose chronology, along with the Par-Tee site chronology, suggests the sites overlapped in their occupations. These data possibly constrain the potential infilling of the former bay near Seaside and affect interpretations of the material record differences between the sites. These findings are consistent with recent Bayesian analyses and chronological studies of previously reported radiocarbon dates applying chronometric hygiene assessments developed for northern Oregon Coast sites [1–12] and support these previous studies’ findings. It demonstrates how AMS dating of museum collections can increase the scientific value of these collections while contributing information to chronologically situate forthcoming and future analyses of the Palmrose and Par-Tee collections. The results of this study suggest the Avenue Q assemblage would benefit from advanced chronological studies while also advocating for the use of short-lived or unambiguous samples in future radiocarbon dating of Oregon Coast sites.
